# The Evolving Landscape of Cancer Stem Cells and Ways to Overcome Cancer Heterogeneity

**DOI:** 10.3390/cancers11040532

**Published:** 2019-04-14

**Authors:** Hiroaki Taniguchi, Yasunori Suzuki, Yukikazu Natori

**Affiliations:** 1 The Institute of Medical Science, The University of Tokyo, 4-6-1, Shirokanedai, Minato-ku, Tokyo 108-0071, Japan; 2 Clinical and Translational Research Center Keio University Hospital, 35 Shinanomachi, Shinjuku-ku, Tokyo 160-8582, Japan; suzukiys@ims.u-tokyo.ac.jp; 3 BioThinkTank Co. Ltd. 4-10-1-E1706 Minatomirai, Nishi-ku Yokohama, Kanagawa 220-0012, Japan; natori0918@gmail.com

**Keywords:** cancer stemness, signaling cascades, in silico approach, cancer heterogeneity, oligonucleotide therapeutics

## Abstract

Cancer stem cells (CSCs) with therapeutic resistance and plasticity can be found in various types of tumors and are recognized as attractive targets for treatments. As CSCs are derived from tissue stem or progenitor cells, and/or dedifferentiated mature cells, their signal transduction pathways are critical in the regulation of CSCs; chronic inflammation causes the accumulation of genetic mutations and aberrant epigenetic changes in these cells, potentially leading to the production of CSCs. However, the nature of CSCs appears to be stronger than the treatments of the past. To improve the treatments targeting CSCs, it is important to inhibit several molecules on the signaling cascades in CSCs simultaneously, and to overcome cancer heterogeneity caused by the plasticity. To select suitable target molecules for CSCs, we have to explore the landscape of CSCs from the perspective of cancer stemness and signaling systems, based on the curated databases of cancer-related genes. We have been studying the integration of a broad range of knowledge and experiences from cancer biology, and also from other interdisciplinary basic sciences. In this review, we have introduced the concept of developing novel strategies targeting CSCs.

## 1. Introduction

Cancer stem cells (CSCs) possess abilities associated with metastasis, resistance to radiation/chemotherapy, and recurrence, and have been detected in several tumor types. CSCs were first identified in leukemia in 1994 [1], and in solid tumors they were first demonstrated in the CD44^+^CD24^−/low^ fraction of breast cancer [2]. Subsequently, CSCs have been detected in the brain, colon, pancreas, and other tissues [3,4]. It has been reported that CSCs express unique surface markers (e.g., CD24, CD26, CD44, CD90, CD133, and CD166), exist in a side-population (SP) fraction that possesses increased Hoechst-33342 efflux capacity, exhibit high aldehyde dehydrogenase-1 (ALDH1) activity, form spheres when cultured in non-adherent conditions [3,5], and show high tumorigenic potential when xenografted as tumor in immunodeficient mice [2,3].

CSCs are thought to be derived from tissue stem or progenitor cells, and/or dedifferentiated mature cells [5]. Therefore, CSCs hijack the signal transduction pathways involved in stemness and the development of normal cells. The Wingless (Wnt), Hedgehog, Notch signals, Runt pathway, Trithorax group (TrxG), and Polycomb group (PcG)-mediated silencing pathways play an important role in the signal transduction pathways for the development of normal stem cells and CSCs. The signal transduction pathways induced by growth factors, such as transforming growth factor-β (TGF-β) and insulin-like growth factor-1 (IGF-1), are involved in the maintenance of stemness of CSCs. Proinflammatory cytokines facilitate CSC generation, suggesting a possible association between CSCs and inflammation. Hypoxia also has critical roles in the regulation of self-renewal in normal cells and CSCs. Moreover, several lines of evidence have suggested that CSCs are formed by a combination of genetic and epigenetic events. Precancerous lesions are thought to be induced by trivial epigenetic alterations caused by inflammation and environmental factors. The accumulation of epigenetic alterations could induce genetic changes in DNA, and the resulting combination of genetic and epigenetic events would therefore promote tumorigenesis and metastasis.

We can speculate on new aspects of the molecular mechanisms of carcinogenesis by using the databases developed for cancer genomics and epigenomics. An informatics approach may offer benefits in the selection of suitable target molecules against CSCs.

This review focuses on the recent developments in research into CSCs and novel therapeutic strategies against CSCs from the perspective of cancer stemness and signaling systems, and introduces many investigations that have used nanoparticle technology to assist therapies targeting CSC. Understanding CSCs from different perspectives will be helpful for the development of novel therapeutic strategies and markers for advanced cancers with malignant phenotype.

## 2. Landscape of Cancer Stem Cells

### 2.1. Properties of Normal Stem Cells and CSCs

CSCs and normal stem cells share many properties such as ‘self-renewal’ and differentiation potentials. Dysregulation of the self-renewal processes may be caused by an increase in the symmetric cell division compared to asymmetric division [6]. As described in the next section, CSCs, but not normal stem cells, possess genetic and epigenetic changes, which lead to the dysregulation of signaling pathways involved in embryonic development, such as Wnt/β-catenin, Hedgehog, Notch, and Hippo signaling, Runt pathway, TrxG, and PcG. Moreover, growth factors (e.g., fibroblast growth factor, TGF-β, and IGF-1) also control the stemness of CSCs. Proinflammatory cytokines (e.g., tumor necrosis factor-α) can facilitate CSC generation, suggesting a possible association with cancer caused by chronic inflammatory diseases, such as chronic pancreatitis, inflammatory bowel disease, and chronic gastritis due to Helicobacter infection. Hypoxia plays an important role in the regulation of self-renewal in CSCs. The effects of hypoxia are mainly mediated by hypoxia-inducible factor (HIF) 1α and HIF2α. MicroRNAs (miRNAs, also known as miRs), small noncoding RNAs that regulate gene expression in a post-transcriptional manner, have been identified as important regulators of CSCs, and miRNAs can alter the signal transduction pathways (e.g., miR-451 inhibiting the Wnt signals and miR-34a inhibiting the Notch signals) [7,8].

In solid tumors, the presence of CSCs was first demonstrated in the CD44^+^CD24^−/low^ fraction of breast cancer cells [2]. CSCs and normal stem cells share cell surface markers (Table 1) and use common stemness-related signaling pathways. However, in normal stem cells, the signaling pathways that maintain stemness are tightly regulated and not mutated. A review divided the 40 published CSC surface markers into three different categories with respect to the marker expression levels in hESCs, adult stem cells, and normal tissue cells [9].

CSCs and normal stem cells exist in a side-population (SP) fraction that possesses increased Hoechst-33342 efflux capacity, exhibits high aldehyde dehydrogenase-1 (ALDH1) activity, and forms spheres when cultured in non-adherent conditions [3]. CSCs and normal stem cells possess the abilities of resistance to chemotherapeutic agents and radiation. These phenotypes might be caused by the ATP-binding cassette (ABC) transporters, antiapoptotic proteins (e.g., BCL2), and enhanced DNA repair systems [10,11]. CSCs also demonstrate high tumorigenic potential when xenografted into immunocompromised mice; specifically, a small proportion of cells in cancer tissues possess the ability to form tumors in vivo. However, normal stem cells do not have the tumor-initiating ability [2,3].

Cancers are composed of heterogeneous combinations of cells because the CSCs are thought to have the ability of multilineage differentiation. However, this ability of CSCs, and not normal stem cells, is impaired, and produces many different cell types in tumor tissues [12].

### 2.2. CSC Formation through Genetic Mutations and Aberrant Epigenetic Changes

Point mutations and dysfunctions of DNA repair in tumors are caused by epigenetic processes. For example, 5-methylcytosine (5 mC) in the gene bodies and coding regions of genes plays an important role in generating the inactivating C to T transition mutations, causing hotspots in somatic cells [48]. The lack of functional expression of MLH1 causes defects in DNA mismatch repair [49]. In addition, many abnormal epigenetic events are induced through genetic abnormalities. Mutations in the Ten-eleven translocation methylcytosine dioxygenase 2 (*TET2*), isocitrate dehydrogenase 1 (*IDH1*), and *IDH2* genes occur in gliomas and leukemia [3,4]. TET proteins generate 5-hydroxymethylcytosine from methylated cytosines and eliminate aberrant DNA methylation in normal cells. TET proteins require α-ketoglutarate as a cofactor; however, the mutant IDH1 and IDH2 change the α-ketoglutarate into the metabolite 2-hydroxyglutarate, which inhibits TET2 [50].

The mouse induced-pluripotent stem cells treated with the conditioned medium derived from mouse Lewis lung carcinoma acquired the characteristics of CSCs, which had a high tumorigenicity in nude mice and a capacity for self-renewal, and expressed the marker genes associated with stem cell properties and an undifferentiated state [51]. The sorted and unsorted fractions of breast cancer cells via CD44, CD24, ALDH, mammospheres, and adherent cell cultures of breast cancer cell lines were analyzed for DNA profiling by array CGH and methylation profiling. There were no genomic differences; however, putative breast CSCs indicated altered methylation levels of several genes compared with the parental tumor cells [52]. After the long-term cell culture of breast tumor cells sorted from pleural effusions for putative breast CSC markers, a CSC population from one pleural effusion sample showed a copy number profile reflecting aberrations in the primary tumor [53]. Validation of the result from whole-genome sequencing by target deep DNA sequencing revealed no genetic changes specifically associated with the breast CSC phenotype. In contrast, the transcriptomic variability by single-cell RNA sequencing distinguished the breast CSCs from the non-breast CSCs by the transcription of 74 genes, which were revealed as breast CSC markers with a high risk of relapse [54]. These phenomena suggest that the genetic changes in tumors are maintained in the cells with CSC and non-CSC phenotype, and epigenetic alternations, including the extracellular environment, confer a survival advantage via plasticity on the cancer cells through rapid transcriptional regulation.

The transient expression of the reprogramming factors (OSKM: Oct3/4, Sox2, Klf4, and c-Myc) in mice induced the transient repression of the pancreatic acinar cell enhancers, such as pancreatitis [55]. Pancreas-specific K-ras activation results in mouse PanIN (pancreatic intraepithelial neoplasia) lesions, but does not proceed to invasive pancreatic cancer [56]. The transient expression of OSKM in *K-ras* G12D mutant mice caused the persistent activation of ERK signaling in pancreatic acinar cells and the rapid formation of pancreatic cancer [55]. Mouse models, expressing an endogenous K-Ras oncogene in pancreatic lineages during embryonic development, developed the full spectrum of PanINs and invasive pancreatic cancer when the K-RasG12V expression was allowed during embryonic development. However, K-RasG12V expression in adult mice does not result in neoplastic development unless they also have chronic pancreatitis [57]. The PRDI-BF1 and RIZ (PR) domain zinc finger protein 14 (PRDM14), known as a transcription factor that maintains pluripotency in stem cells via epigenetic regulations, reduced the methylation of proto-oncogene and stemness gene promoters in cancer cells. Moreover, strong PRDM14 binding sites coincided with promoters containing both H3K4me3 and H3K27me3 histone marks, which are called ‘‘bivalent marks’’ observed in undifferentiated cells [58]. PRDM14 is overexpressed in pancreatic cancer tissues and regulates the cancer stem-like phenotypes in pancreatic cancer cells [59]. Increased levels of PRDM14 were observed in mice models of chronic pancreatitis induced by cerulein, but this model does not result in neoplastic development [60]. Collectively, the pancreatic ductal adenocarcinoma stem cells could form by a combination of genetic (e.g., somatic *K-Ras* mutations) and nongenetic (e.g., chronic inflammation and reprogramming factors) events. 

## 3. Therapeutic Strategy against CSC

### 3.1. Selection of Suitable Target Molecules against CSCs via In Silico Approach

Publicly accessible databases for cancer genomics and epigenomics have been developed and have improved scientific discovery, and these databases can be exploited without significant practical laboratory experience, in other words, an “In Silico Approach”. For example, The Cancer Genome Atlas (TCGA) is a National Cancer Institute project that profiles the genetic mutations responsible for different tumor types using genomic sequencing and bioinformatics (https://cancergenome.nih.gov/) [61]. The Catalogue Of Somatic Mutations In Cancer (COSMIC) database collects somatically-acquired mutations found in human cancers derived from the TCGA and the International Cancer Genome Consortium (ICGC), and has been curated by expert scientists in a large numbers of scientific publications (https://cancer.sanger.ac.uk/cosmic) [62]. MethHC is the human pan-cancer methylation database focused on the DNA methylation of human diseases, such as DNA methylation, microRNA expression, and the correlation of methylation and gene expression from TCGA (http://methhc.mbc.nctu.edu.tw/php/index.php) [63]. To collect the phenotypic and clinical information on variants across the genome, the CIViC (https://civicdb.org/home) [64], ClinGen (https://www.clinicalgenome.org/) [65], and ClinVar (https://www.ncbi.nlm.nih.gov/clinvar/) [66] databases allow a curated collaborative approach to maintain up-to-date genomic information. Even though these databases are not focused on genetic or epigenetic abnormality of CSCs, we can apply these web resources to cancer genomics and epigenomics research based on the cancer types, signaling systems in cancer, and our experience.

To select the suitable target molecules for CSCs, we also have to explore the landscape of CSCs from the perspective of cancer stemness. Recently, a database of CSCs has been constructed. The Cancer Stem Cells Therapeutic Target Database (CSCTT) contains 135 proteins that are potential targets of CSCs, and has 213 documented therapeutic methods for CSCs, including 118 small molecule and 20 biotherapy methods (http://www.csctt.org/) [67]. Another group developed a CSCs database (CSCdb), which includes the information on 74 marker genes, 1769 CSC-related genes, and 9475 functional annotations about 13 CSC-related functions derived from approximately 13,000 articles (http://bioinformatics.ustc.edu.cn/cscdb) [68].

**Figure 1:**
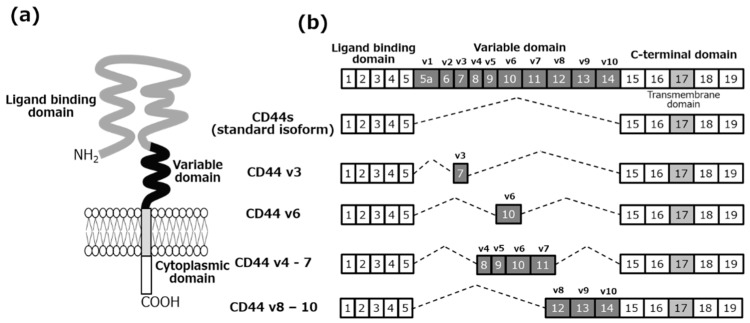
CD44 and CD44 splice variants. (**a**) CD44 is a transmembrane glycoprotein. CD44 consists of the extracellular domain, the transmembrane domain, and the cytoplasmic domain. The ligand binding/extracellular domain interacts with hyaluronic acid, osteopontin, chondroitin, collagen, and fibronectin. The CD44 cytoplasmic domain mediates transcription. (**b**) The *CD44* gene consists of 20 exons. *CD44* gene, exons 1–5 and 16–20 produce the standard isoform of CD44, referred to as the CD44 standard (CD44s). The remaining exons 6–15 are alternatively spliced and assembled with the ten exons contained in the CD44 standard isoform and are referred to as the CD44 variant isoforms (CD44v).

It is important to understand the usage of splice (mRNA) variants according to the cancer cell fate and cancer phenotype, because it has been reported that splicing is deregulated in cancer. Not only the signaling cascades, but also the alternative splicing in CSCs, has the possibility to be targeted with oligonucleotide therapeutics. For example, CD44, a non-kinase transmembrane single-chained glycoprotein, is overexpressed in several cell types including cancer stem cells. In the *CD44* gene, the exons 1–5 and 16–20 produce the standard isoform of CD44, referred to as the CD44 standard (CD44s). The remaining exons 6–15 are alternatively spliced and assembled with the ten exons contained in the CD44 standard isoform and are referred to as the CD44 variant isoforms (CD44v) [69] (Figure 1). The CD44 splice variants are differentially expressed in both the normal and cancer cells, e.g., CD44v8-10 in pancreatic cancers [70] and CD44v6 in colorectal cancer including CSCs, which is a useful marker of tumor prognosis [71,72]. CD44v8-10 stabilizes a cystine-glutamate transporter, xCT, in the plasma membrane of cancer cells, resulting in the promotion of glutathione synthesis and the GSH-dependent antioxidant system in cancer cells [73]. The SNP located within intron 3 of *POU5F1/OCT4* is associated with the recurrence of prostate cancer. This SNP might affect mRNA splicing by altering the exonic splicing enhancer binding. The *POU5F1* gene has alternative splicing variants: OCT4A, OCT4B, and OCT4B1. OCT4A regulates the stem cell pluripotency and self-renewal, as a transcriptional factor. However, OCT4B and OCT4B1 are thought to be related to the stress response and anti-apoptotic properties [74]. To explore the alternative splicing based on TCGA samples from 33 tumors, TSVdb (http://www.tsvdb.com) was developed as a web-based tool, which has integrated the clinical data, gene expression, usage of exons/junctions, and splicing patterns [75]. To accomplish effective cancer therapy, we should focus on the variants of cancer stem cell markers, as shown in Table 2, as the number of the variants reported has been increasing dramatically. The variants are supposed to be due to posttranslational modifications.

### 3.2. Ongoing Challenges of Treating CSC with Several Modalities

**Figure 2:**
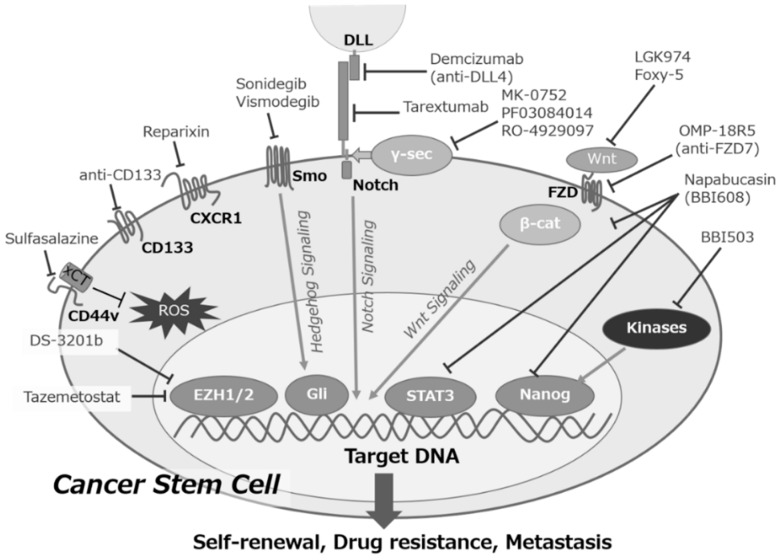
Aberrant signal transduction pathways in CSCs and therapeutic agents targeting CSCs. Signal transduction pathways play important roles in self-renewal, drug resistance, tumor recurrence, and distant metastasis in CSCs, such as CD133, CXCL8, Hedgehog, Notch, and Wnt signaling, and the transcription factors β-catenin (β-cat), signal transducer and activator of transcription 3 (STAT3), EZH2, and Nanog. After interaction with xCT, a CD44 variant (CD44v) enhanced the capacity for glutathione synthesis and the defense against reactive oxygen species (ROS). DLL, delta-like ligand; γ-Sec, γ-Secretase; FZD, Frizzled; Smo, Smoothened.

Conventional chemotherapies are effective to eliminate much of the tumor tissues consisting of non-CSCs. The effectiveness of conventional therapies against cancers depends on metastasis, resistance to radiation/chemotherapy, and recurrence, which are all thought to be caused by cancer stem cells (CSCs). Chemo- and radio-therapies have the ability to activate cellular stress response and to induce stemness characteristics in non-CSCs. These therapies then lead to the enrichment of a CSC subpopulation with higher resistance to these therapies [76]. For example, CD133/CXCR4 double-positive cancer cells were enriched in chemotherapeutic-resistant colorectal cancer [77]. Therefore, new therapeutic options against CSCs must be developed (Figure 2).

Several approaches have been adopted to target the surface markers of CSCs, and the signal transduction pathways involved in CSCs, such as Wnt, Hedgehog, Notch, and STAT3. For example, sulfasalazine (SSZ), an inhibitor of xCT in the CD44v-positive CSCs, combined with cisplatin and anti-CD44 antibodies, have been effective in gastric cancer, lung cancer, and acute myeloid leukemia due to the reduced intracellular glutathione (GSH) levels and inducing terminal differentiation of CSCs [78,79,80]. The treatment with an antibody against another CSC marker, CD133, proved highly effective for eliminating several type of grafted tumors in vivo [81], and the bi-specific antibodies initiating T cell responses and immunotoxin against CD133 have recently been developed [82]. Chemokine receptor 1 (CXCR1), a receptor for CXC ligand 8 (CXCL8), which is a proinflammatory chemokine, is one of the actionable receptors expressed in breast cancer stem cells. The inhibitor of CXCR1/2, reparixin, has been effective in human breast cancer xenografts, and a phase Ib clinical trial has been completed in patients with metastatic breast cancer [83].

The two Smo inhibitors, Sonidegib and Vismodegib, as Hedgehog pathway inhibitors, have been effective in basal cell squamous carcinoma; however, there was no clinical benefit in combining vismodegib with the first-line regimen against metastatic colorectal cancers (CRCs) in the phase II trial [84]. The small molecule, LGK974, an inhibitor of the O-acyltransferase porcupine that acetylates Wnt proteins, and Foxy-5, a Wnt-5a-mimicking hexapeptide, are in a phase I trial as metastasis-inhibiting agents for CRC. [85,86]. Another approach targeting the Wnt pathway with an anti-Frizzled 7 monoclonal antibody, OMP-18R5, reduced the tumor-initiating cell frequency, and exhibited synergistic activity with the standard-of-care chemotherapeutic agents [87]. MK-0752, a gamma secretase inhibitor, which is an inhibitor of the Notch pathway, enhanced the efficacy of docetaxel in the preclinical studies for breast CSCs [88]. The humanized monoclonal antibodies, demcizumab (targeting Notch ligand, delta-like-4) and tarextumab (targeting the Notch-2/3 receptors), have been evaluated in combination with standard chemotherapy in phase II trials for metastatic pancreatic cancer. The phase III trials of napabucasin (BBI608), an orally-administered drug targeting phospho-STAT3, Nanog, and β-catenin, for advanced CRC and gastric cancer, was closed due to the poor outcome. Tazemetostat, a selective inhibitor of EZH2, showed antitumor activity in patients with refractory B-cell non-Hodgkin lymphoma and advanced solid tumors without severe toxicities [89]. The phase I clinical study of DS-3201b, an EZH1/2 dual inhibitor, indicated that the overall response rate was 58.8% for patients with relapsed or refractory non-Hodgkin lymphoma [90].

Epigenetic abnormalities, such as DNA methylation, histone modification, and miRNA, can contribute to the progression of CSCs. Azacitidine and aza-deoxycytidine (aza-dC), the DNA methyltransferase inhibitors, have already been integrated into therapy for AML and myelodysplastic syndromes. Histone deacetylases (HDACs) play an important role in the epigenetic machinery regulating gene expression, act as oncogenes in several cancers, and can modulate chemotherapeutic resistance in hematologic neoplasms. Treatment with a HDAC inhibitor, combined with imatinib mesylate, suppressed the CML stem cells [91]. Moreover, the HDAC inhibitors can reprogram the differentiated triple-negative breast cancer cells via the pentose phosphate pathway, which is targeted by the inhibition of glucose-6-phosphate dehydrogenase [92].

### 3.3. Nanoparticles for Therapeutic Drug Targeting CSC

As recent approaches, nanoparticles (NPs) effectively target cancer cells that are difficult to treat, such as CSCs. The advantage of NPs is mainly derived from their high surface-to-volume ratio compared with their bulk counterparts. Moreover, targeting agents are able to attach to the surface of NPs for molecular recognition [93]. Consequently, NPs can control drug delivery through their size, charge, and surface molecules, and more efficiently release drugs via NPs structure. Currently, new CSC treatment using NPs have targeted specific markers or signaling pathways involved in CSC function and maintenance [94]. The NPs were prepared with biodegradable methoxy poly (ethylene glycol)-block- poly (D, L-lactic acid) (mPEG-b-PLA) and loaded with doxorubicin (NPDOX) or decitabine (NPDAC), an inhibitor of DNA methylation. NPDAC significantly reduced ALDH-positive stem-like population in malignant breast cancer through the inhibiton of cancer cell growth [95].

Liposomal NP (LNP) incorporated paclitaxel or coated anti-CD44 antibody loaded with a suicide gene or doxorubicin induced therapeutic effects in CD44-positive metastatic ovarian cancer cells or hepatocellular carcinoma cells [96,97]. Hyaluronic acid-coated chitosan NPs loaded with 5-fluorouracil (5-FU)/oxaliplatin enhanced cytotoxicity compared with either 5-FU or oxaliplatin alone in human colorectal cancer cells, which overexpressed CD44 [98,99]. Anti-CD90 antibody-mediated water-soluble cadmium selenide core nanocrystals loaded with photosensitizers target the CD90-positive leukemia CSCs and sensitized leukemia CSCs to UV irradiation [100].

Polymeric NP encapsulating curcumin significantly reduced clonogenic growth and CD133-positive stem-like population in malignant brain tumors [101] and induced apoptotic effects in cisplatin-resistant ovarian cancer cells through the suppression of the Wnt/β-catenin signaling component [102]. Salinomycin-loaded PEGylated poly (lactic-co-glycolic acid) NPs (SAL-NP) conjugated with CD133 aptamers (Ap-SAL-NP) reduced the proportion of CD133+ osteosarcoma cells [103]. 5-FU loaded NPs can effectively inhibit the peritoneal dissemination of colorectal cancer cells overexpressing Wnt/β-catenin signaling components [104].

Hydrophobic drugs-conjugated with mesoporous silica NPs (MSNPs) enhanced cytotoxicity by selective targeting of Notch signaling in various cancer cell types, such as cervical and breast cancer cells [105]. Jagged1 (Notch ligand) siRNAs-loaded chitosan NPs inhibited ovarian cancer [106]. Gli antagonist (HPI-1)-conjugated polymeric nanoparticle (NanoHHI) significantly suppressed the growth and invasion of CD133-positive cells in hepatocellular carcinoma [107]. Anthothecol (a limonoid derived from plants) conjugated with poly-lactide-co-glycolide acid (PLGA) NPs (Antho-NPs) effectively reduced the ability of cell proliferation and colony formation, and induced apoptosis in pancreatic CSCs through the modulation of Hh signaling [108].

Polyethyleneimine/polyethylene glycol-conjugated MSNPs possessed a high-loading capacity and exhibited pH-dependent release of LY364947, a small molecule TGF-β inhibitor, which significantly decreased xenograft tumor size of breast cancer cells compared with LY364947 alone [109]. Cationic lipid-assisted polymeric NPs combined with siRNA and LY36494 indicated remarkable tumor regression and a notable decrease in CSC frequency [110]. Gold NPs (AuNPs) combined with TGF-β1 via S–Au binding suppressed the TGF-β signaling pathway, and attenuated the immunosuppressive function of TGF-β [111].

PRDM14 confers CSC phenotypes in breast cancer cells, such as resistance to chemotherapy and side population. In-vivo tumorigenic potential and sphere-forming ability was greatly facilitated in PRDM14-overexpressing cancer cells, although these findings did not fully support the role of PRMD14 in cancer stemness [58]. When double-stranded RNA/DNA chimera [112] against PRDM14 combined with calcium phosphate hybrid micelles [113] was administered intravenously as NPs, the growth of the grafted breast tumor was decreased compared with negative control; further decreases were observed when the mice were additionally treated with docetaxel [58].

## 4. Conclusions

CSCs have been identified from a wide variety of human tumor types, such as breast, lung, prostate, colon, brain, and head and neck tumors, pancreatic cancer, and lymphoma/leukemia. CSCs are characterized by their abilities of self-replication, plasticity, therapeutic resistance, and distant metastasis. CSCs have been identified and isolated, based on the expression of specific molecules, such as CD24, CD34, CD44, CD133, and aldehyde dehydrogenase (ALDH), and are able to regenerate tumor mass from a small number of cells when implanted into immunodeficient mice.

We are able to select the target genes of CSCs from publicly-accessible curated databases for cancer-related genetic and epigenetic alterations. These databases have been constructed via curation by scientific specialists and a large amount of data processed by next generation sequencer (NGS) using clinical tumor samples. Moreover, as the CSCs are assumed to be a rare population of tumor cells, or at least not to the majority of cells, we are able to apply next generation sequencing to the analysis of cancer-related genetic and epigenetic alterations after the single cell isolation or cell sorting by flow cytometer, to avoid contamination with the non-CSCs and stromal cell around the tumor cells. However, the properties of CSCs, especially the cell surface antigens, metabolic pathways, and epigenomic status, might be changed easily and instantaneously due to environmental factors affecting the cancer tissues. Therefore, to investigate the properties of CSCs, it is important to validate them using immunohistochemical (IHC) analysis of cancer tissue slices handled by adequate methods or to analyze the primary cultured cells or tumor spheroids for experiments of CSCs with clinical samples. However, it is not clear whether we are able to confirm the properties of CSCs when we analyze the primary cultured cells or tumor spheroids without the interstitial tissues under the artificial culture conditions. IHC analysis also has severe problems caused by the quality and the specificity of antibodies, and the standardization of slide scoring.

At present, we are able to screen target genes of CSCs from the perspective of cancer stemness and signaling systems, based on publicly-accessible curated databases for cancer-related genetic and epigenetic alterations. After the candidate genes are selected, they should be analyzed in tissue slices derived from patients with cancer by using IHC techniques and in the primary cultured cells or tumor spheroids established from clinical cancer tissues. The oligonucleotide therapeutics or bi-specific antibodies are ideally suited for the purpose of CSC elimination because they simultaneously suppress several molecules involved in the signal transduction in CSCs, and for oligonucleotide therapeutics, a particular mRNA variant related to the malignant cancer phenotype can be identified with flexibility based on the sequence information only.

Until recently, we focused on several types of molecules based on empirical approaches and attempted to treat some cancers using target molecules in cell surface receptor type kinases by using the conventional small molecules and/or antibodies. Now, the time has arrived to select the target molecules in various types of cancers by using publicly-accessible curated databases, and perform a comprehensive analysis of CSCs based on analysis of both the genetic and epigenetic changes, including mRNA variants. Consequently, we will be able to proceed with the development of innovative treatment methods for cancers with oligonucleotide therapeutics and modified antibodies or peptides, in combination with the conventional therapies.

## Figures and Tables

**Table 1:** Representative unique markers of cancer stem cells.

Tumor Type	Representative Unique Markers	References
**Acute Myeloid leukemia**	*CD34*^+^ CD38^−^	[13]
**Acute lymphoblastic leukemia**	*CD34*^+^*CD10*^−^ CD19^−^	[14]
**Multiple myeloma**	CD138^+^	[15]
**Brain**	*CD133* ^+^	[16]
	CD15^+^	[17]
**Head and neck**	CD44^+^	[18]
**Breast**	EpCAM^+^ CD44^+^ *CD24*^−/low^ Lineage^−^	[2]
	CD44^+^ *CD24*^−^ ALDH1^+^	[19]
**Lung**	*CD133^+^*	[20]
	Sca-1^+^ CD45^−^ PECAM^−^ *CD34*^+^	[21]
**Esophagus**	*CD90* ^+^	[22]
	*T75NTR(CD271)* ^+^	[23]
	TACC3^+^	[24]
	*ICAM1* ^+^	[25]
	CD44^+^ ALDH1^+^	[26]
**Gastric**	CD44^+^	[27]
**Colorectal**	*CD133*^+^ CD44^+^ ALDH1^+^	[28]
	EpCAM^+^ CD44^+^ *CD166*^+^	[29]
	CD44^+^ *CD24*^+^	[30]
	*Lgr5* ^+^	[31]
	ALDH^+^	[32]
**Colon (metastatic)**	*CD133* ^+^ *CD26* ^+^	[33]
**Liver**	*CD133* ^+^	[34]
	*CD133* ^+^ *CD49f* ^+^	[35]
	*CD90*^+^ CD45^−^	[36]
	*CD13* ^+^	[37]
	*EpCAM* ^+^	[38]
**Pancreas**	*CD133*^+^ CD44^+^ *CD24*^+^ EpCAM^+^	[39]
	*CD133* ^+^ *CXCR4* ^+^	[40]
**Ovary**	CD44^+^ *CD117*^+^	[41]
	*CD133* ^+^	[42]
**Prostate**	*CD133*^+^ CD44^+^ α2β1high	[43]
	Sca-1^+^	[44]
**Bladder**	CD44^+^	[45]
**Melanoma**	ABCB5^+^	[46]
**Skin**	*CD34* ^+^	[47]

Surface markers expressed on both CSC and normal stem cells are written in italics [9].

**Table 2:** Major cancer stem cell markers and their variants.

CSC Markers	# of Variants	CSC Markers	# of Variants
**ABCG2**	10	**CD166 (ALCAM)**	4
**ABCG5**	9	**CD271 (NGFR)**	1
**ALDH1**	18	**DCLK1**	8
**ANTXR1**	6	**DDX4**	7
**BMI1**	1	**DNAJB8**	2
**CDCP1**	5	**EGFRv** **Ⅲ** **(EGFR)**	9
**COLEC10 (CLL1)**	3	**EpCAM**	1
**CD19**	5	**GD2 (glycosphingolipid)**	1
**CD24**	6	**LGR4**	2
**CD26 (DPP4)**	2	**LGR5**	3
**CD34**	2	**NANOG**	2
**CD44**	22	**NANOG1**	1
**CD47**	5	**NANOGP8**	2
**CD90 (THY1)**	3	**NOTCH1**	5
**CD105 (ENG)**	3	**OCT4 (POU5F1)**	1
**CD110 (MPL)**	2	**NGFR (p75NTR)**	1
**CD117**	8	**Podoplanin (PDPN)**	7
**CD123 (IL3RA)**	8	**SOX2**	1
**CD133 (PROM1)**	22	**TIM3 (HAVCR2)**	1

NCBI search keyword: biomol_rna [properties] AND “Homo sapiens” [porgn]. As of July 2018, total number of variants in Refseq and mRNA of NCBI Nucleotide is 113,766.
